# Integrating vector control within an emerging agricultural system in a region of climate vulnerability in southern Malawi: A focus on malaria, schistosomiasis, and arboviral diseases

**DOI:** 10.1016/j.crpvbd.2023.100133

**Published:** 2023-07-13

**Authors:** Christopher M. Jones, Anne L. Wilson, Michelle C. Stanton, J. Russell Stothard, Federica Guglielmo, James Chirombo, Lindiwe Mafuleka, Rose Oronje, Themba Mzilahowa

**Affiliations:** a Department of Vector Biology, Liverpool School of Tropical Medicine, Liverpool, UK; b Tropical Disease Biology, Liverpool School of Tropical Medicine, Liverpool, UK; c Malawi-Liverpool Wellcome Trust, Blantyre, Malawi; d African Institute for Development and Policy (AFIDEP), Nairobi, Kenya; e Malaria Alert Centre, Kamuzu University of Health Sciences, Blantyre, Malawi

**Keywords:** Malaria, Schistosomiasis, Irrigation, *Anopheles*, Agriculture, Vector control

## Abstract

Infectious diseases are emerging at an unprecedented rate while food production intensifies to keep pace with population growth. Large-scale irrigation schemes have the potential to permanently transform the landscape with health, nutritional and socio-economic benefits; yet, this also leads to a shift in land-use patterns that can promote endemic and invasive insect vectors and pathogens. The balance between ensuring food security and preventing emerging infectious disease is a necessity; yet the impact of irrigation on vector-borne diseases at the epidemiological, entomological and economic level is uncertain and depends on the geographical and climatological context. Here, we highlight the risk factors and challenges facing vector-borne disease surveillance and control in an emerging agricultural ecosystem in the lower Shire Valley region of southern Malawi. A phased large scale irrigation programme (The Shire Valley Transformation Project, SVTP) promises to transform over 40,000 ha into viable and resilient farmland, yet the valley is endemic for malaria and schistosomiasis and experiences frequent extreme flooding events following tropical cyclones. The latter exacerbate vector-borne disease risk while simultaneously making any empirical assessment of that risk a significant hurdle. We propose that the SVTP provides a unique opportunity to take a One Health approach at mitigating vector-borne disease risk while maintaining agricultural output. A long-term and multi-disciplinary approach with buy-in from multiple stakeholders will be needed to achieve this goal.

## Highlights

• Vector-borne disease incidence is associated with irrigation.
• The interaction between vectors, hosts and parasites in agricultural systems is context specific.
• An emerging agricultural landscape in southern Malawi may reshape vector-borne disease transmission patterns.
• Understanding and controlling vector-borne disease in the Lower Shire Valley will need a One Health approach.

## Graphical abstract

**Figure:**
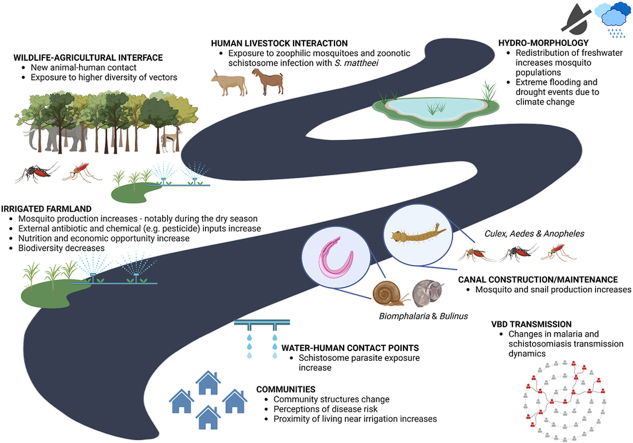

## 1 Background

Agricultural expansion and infectious diseases interact in a myriad of ways. The intensification of agricultural practices through irrigation redistributes freshwater, reduces plant and insect biodiversity, increases contact between domestic livestock and humans, and requires an increase in pesticide and antibiotics use (Rohr et al., 2019); all of which can, depending on the context, promote infectious disease risk. There is a well-established increased risk of vector-borne diseases such as malaria and schistosomiasis in irrigated farmland (Steinmann et al., 2006; Kibret et al., 2021; Chan et al., 2022) and this threat is particularly acute in low middle income countries (LMICs) where high rates of agricultural development overlap with the presence of endemic and emerging infections.

To improve food security, there is renewed interest in large-scale government-led and donor-funded irrigation schemes across sub-Saharan Africa (Higginbottom et al., 2021). Irrigation can provide a reliable and secure water source to small-holder farmers, particularly during seasonal shortages. Furthermore, access to irrigated land expands commercial opportunities, diversifies cropping regimes, and allows farmers to engage in multiple cropping seasons. The potential health, economic, and societal benefits of irrigation to rural communities are widely acknowledged (Ward et al., 2016), although historically, such large schemes have underperformed in delivering their intended irrigated land areas across Africa (Higginbottom et al., 2021).

The south-east African region sits within a climate transition zone (Siderius et al., 2021), in which the El Nino-Southern Oscillation (ENSO) and the Indian Ocean Dipole (IOD) drive highly variable annual rainfall patterns (the standard deviation of annual rainfall is ∼200 mm). Most climate models project an increase in interannual rainfall variability, and it is against this backdrop that investments in long-term irrigation infrastructure in south-east Africa are being developed to absorb such future weather variations. The nature of these schemes varies, ranging from the rehabilitation of existing irrigation (e.g. The Lower Nzoia Irrigation Project (Tanzania)) to the construction of large dams to supply irrigation water (Mwache Multipurpose Dam Project (Kenya) and Mwomboshi Dam, (Zambia)).

## 2 Food security in Malawi and The Shire Valley Transformation Project

The projected population of Malawi for 2050 is currently estimated at 33 million, nearly double the 17.5 million recorded in the latest 2018 census (National Statistical Office of Government of Malawi, 2019). Feeding a rapidly expanding population on the same area of land will be one of the country’s most significant challenges. Around 80% of the rural population of Malawi currently depend on rainfed subsistence farming, with a heavy reliance on maize. Rainfall in the south-east African region is highly variable year-to-year, making harvests vulnerable (Siderius et al., 2021). Subsistence farming within Malawi is, therefore, extremely vulnerable, and this is especially true for maize, which requires at least 500 mm of rainfall a year (Stevens and Madani, 2016). This has been further exacerbated by recent extreme weather events, such as Cyclone Idai in 2019, Storm Ana in 2022 and, most recently, Storm Freddie in 2023 (Otto et al., 2022).

**Fig. 1:**
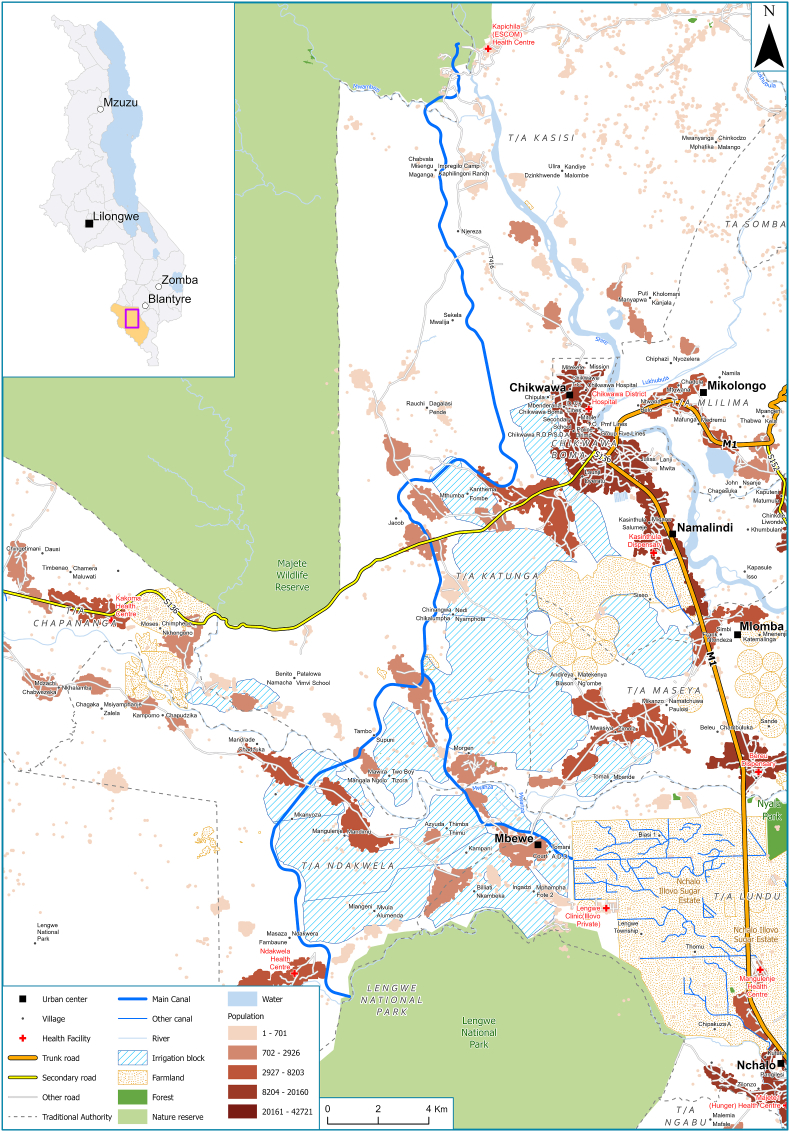
A map showing the main canal and extent of proposed irrigation under the first phase of the Shire Valley Transformation Project (SVTP) in the lower Shire Valley of southern Malawi. The site of the SVTP within Malawi is illustrated in the inset. Population estimates, existing irrigated farmland and nature reserves are also shown.

Approximately 25% of in Malawi (> 400,000 ha) is under irrigation, and the Government of Malawi aspires to double this figure by 2035 (Malawi Government, 2016). Part of realising this target is the development of the Shire Valley Transformation Project (STVP), a World Bank funded project to transform over 40,000 ha in the lower Shire Valley of southern Malawi into irrigated land. Gravity-fed flood irrigation will flow from the Kapichira Dam adjacent to the Majete Wildlife Park *via* 140 km of newly constructed canals to support a series of new farmland parcels. The scheme is scheduled to complete in the early part of the 2030 decade, in a series of two overlapping phases passing through the districts of Chikwawa and Nsanje. The extent of proposed irrigation as part of the first phase of the SVTP is shown in Fig. 1. The SVTP will additionally promote natural resource management in four protected wildlife reserves and a wetland area (Elephant Marsh).

**Fig. 2:**
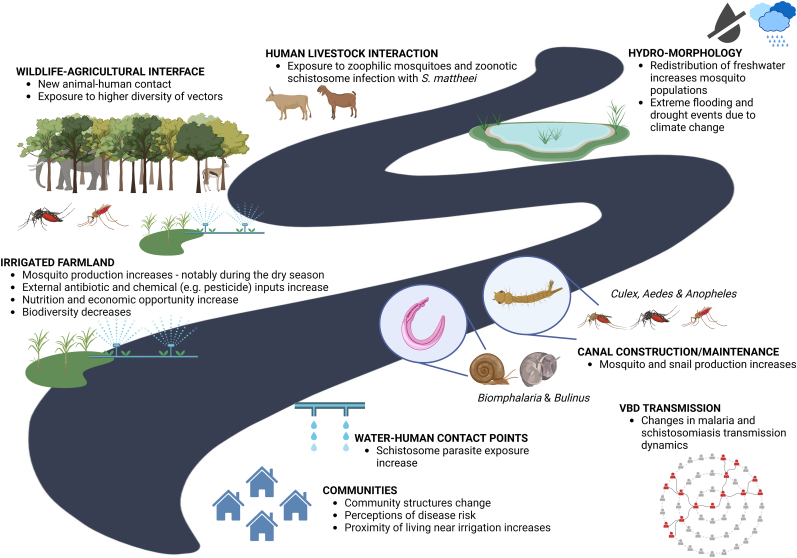
Schematic showing the possible effects from the Shire Valley Transformation Project (SVTP) on vector-borne disease in the lower Shire Valley. Note that these are hypothesised impacts based on our understanding of the interaction between irrigation and vector-borne disease. Created in BioRender.com.

In Malawi, where the agricultural sector depends almost entirely on rain-fed subsistence farming and employs 85% of the population, developments in farming systems and practices can benefit society, the economy, and nutrition. That said, converting over 40,000 ha of land in the lower Shire Valley will have an impact on infectious disease transmission that warrants further investigation, especially given the current endemic and epidemic potential of various communicable diseases. Some of the possible effects on vector-borne disease transmission following the introduction of large-scale irrigation are summarised in Fig. 2. These are briefly discussed below.

**Fig. 3:**
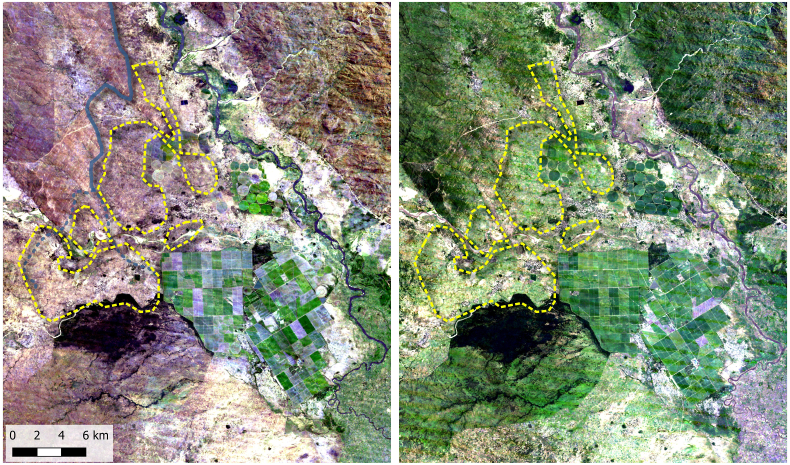
Satellite imagery of the Chikwawa area in the dry (left panel) and rainy (right panel) season with the proposed Shire Valley Transformation Project (SVTP) outline (yellow dashed line). The Illovo sugar estate sits to the bottom right of the image giving a snapshot of what irrigated land in the area will look like once irrigation from the SVTP is in full operation.

The lower Shire Valley is classified as a semi-arid dryland. Agricultural production in this region is closely correlated with rainfall. The lack of surface water during the dry season is particularly acute; yet, the 12,000 ha Illovo sugar estate situated on the banks of the Shire River demonstrates the agricultural potential of the land when continuous (pumped) irrigation is available. These sugar fields are a snapshot of what the future landscape will look like once the SVTP is complete (Fig. 3).

## 3 The potential impact of agricultural land conversion on vectors, parasites, and hosts in the lower Shire Valley

Landscape features partially influence the density, behaviour, and distribution of insect vectors (Lambin et al., 2010). At the biological level, the link between irrigation and mosquito and snail abundance is unequivocal and the proximity of living near large scale irrigation dams is a well-known risk factor for malaria and schistosomiasis throughout the continent (Steinmann et al., 2006; Kibret et al., 2021). The population dynamics of insect vectors which rely on standing or flowing water for part (e.g. mosquitoes) or all (e.g. schistosome intermediate snail hosts) of their life-cycle shift with the availability of aquatic habitat. For instance, mosquito populations undergo a bottleneck during the dry season in the absence of surface water. The creation of irrigated farmland supplied by a network of canals provides additional habitat for endemic vectors, boosting population numbers, lengthening the season of habitat suitability, and consequently, extending and potentially amplifying disease transmission depending on parasite rates. Furthermore, the increased connectivity of vector populations reduces the risk of local extinction from stochastic population processes (McCormack et al., 2019).

The main malaria vector associated with irrigated land in East Africa is *Anopheles arabiensis* (a member of the *Anopheles gambiae* species complex). Typically, *An. arabiensis* mosquitoes lay eggs in transient shallow water habitats. In Ethiopia, malaria incidence and *An. arabiensis* densities are consistently higher in proximity to small and large-scale irrigation dams as well as canal-based schemes (Kibret et al., 2017; Demissew et al., 2020). In a meta-analysis of 36 entomological studies comparing rice and non-rice growing areas, human biting rates were consistently higher in rice-growing villages (with *An. gambiae* (*s.l.*) being the dominant vector in 35 studies) (Chan et al., 2022). In villages situated inside the Illovo sugar estate in Chikwawa, *An. arabiensis* is the predominant outdoor host-seeking vector during the dry season (Zembere et al., 2022). Historically, the lower Shire Valley experiences high malaria transmission levels (Chirombo et al., 2020). Parasitological prevalence data suggest, however, that transmission has fallen since the implementation of routine mass distribution of insecticide-treated nets (Chipeta et al., 2019) (for example, the predicted average reduction in parasite prevalence in children aged between 2 and 10 years-old in Chikwawa district was 54.8% between 2010 and 2017) and a recent cluster randomised controlled trial in villages surrounding the Majete Game Reserve reported low transmission rates in the area (McCann et al., 2021). The introduction of large-scale agriculture could provide the ideal conditions for a resurgence in malaria rates in the region (Thomas et al., 2012). The combination of emergent vegetation and transient shallow water bodies is the ideal habitat for *An. gambiae* (*s.l.*) mosquitoes and populations tend to thrive in grassland habitat. The pollen from cultivated lands is a key nutrient source for *Anopheles* larvae and there is evidence that plant volatiles attract gravid female *Anopheles* mosquitoes to grass pollen rich environments as an evolutionary adaptation (Wondwosen et al., 2018). At the epidemiological level, malaria incidence is often higher in proximity to agricultural schemes, although disentangling the biological (irrigation, attraction to crops) and socio-economic aspects (e.g. more populous area) of this relationship is difficult (Ijumba and Lindsay, 2001), but water also plays a central role in linking ecologies with institutions and infrastructures (Krause and Harris, 2021), and long-term implementations must make of sustainability one of their pillars. Multispecies and more-than-human approaches are required to understand how land-use changes associated with irrigation influence the interaction between people, wildlife, and insect vectors. The politics of water provision, in particular when tied to land management, define communities as household, kin, ethnic groups through moral sentiments of reciprocity (Peters, 2010). However, competition can arise when demand outweighs resources (Mandala, 1984) and long-standing relationships are affected, as might become the case with community resettlement: where land ownership is linked to inheritance, which can be both patrilineal (from fathers to sons) and matrilineal (from mothers to daughters), resettlement and land re-distribution can be expected to affect intrahousehold decision-making (Meijera et al., 2015), social mobility, and labour. Technological advancements, social change, and health - human and environmental - can no longer be thought as separate realities.

The full range of zoonotic and anthroponotic pathogens circulating in the lower Shire Valley is currently unknown. A consequence of converting to monocultural farmland is a reduction in biodiversity and a fragmentation of the landscape (Davis et al., 2023). The risk of zoonotic disease emergence is heightened in areas of reduced biodiversity with vectors restricted to feeding on the same primary reservoirs of disease and boosted by the favourable and abundant farmland ecosystem. The catchment area for the SVTP will encroach or sit adjacent to four natural forest and woodland protected habitats: Majete Wildlife Reserve (77,754 ha), Lengwe National Park (77,578 ha), and Mwabvi (33,193 ha) and Matandwe (28,915 ha) Forest Reserves. Majete and Lengwe are home to a diverse range of mammals and birds. Pathogen spillover occurs at locations at the interface where animals (wildlife and domestic) and humans frequently converge (Plowright et al., 2017)*.* The proximity of a host to an ecosystem boundary is expected to mediate the rates and risks of these spillover events, and host species richness tends to be higher at the boundary zone where different species mix.

The evidence for other mosquito-borne pathogens in Malawi is scarce but evidence from neighbouring countries suggests that some level of arboviral transmission is likely. Arboviruses are difficult to diagnose clinically due to the presentation of non-specific symptoms (which may overlap with malaria) and lack of diagnostic capacity. Seroprevalence studies in human samples from Zambia and Mozambique indicate circulation of chikungunya, Zika, dengue, and Mayoro viruses in the population (Abílio et al., 2018; Chisenga et al., 2020) but the vectors involved remain poorly understood. It is likely that similar arboviral antigens circulate in human populations in Malawi; yet, no evidence for this is currently available. Certainly, the *Aedes, Culex* and *Anopheles* vectors of arboviruses are widespread in Malawi (Maekawa et al., 2021) but their ecology, interaction with human and animal hosts and distribution is relatively unexplored.

Whilst there is a National Control Programme for schistosomiasis in Malawi, disease transmission is highly focal, with considerable variation across the country both in time and space (Makaula et al., 2014). With current bottlenecks in disease surveillance coverage, many locations remain poorly known, or unsampled, with epidemiological investigations fragmented. Maps of the distribution of intermediate snail hosts are very rudimentary and it has recently come to light, through the application of molecular DNA markers, that species diversity within the genus *Bulinus* has been underestimated considerably (Alharbi et al., 2022). Both forms of schistosomiasis are present in the lower Shire Valley: autochthonous transmission of urogenital schistosomiasis is proven whilst local transmission of intestinal schistosomiasis is enigmatic (Poole et al., 2014; Kayuni et al., 2020). The latter arises from the peculiar absence of its intermediate snail host species although its keystone intermediate snail host species *Biomphalaria pfeifferi* has been increasing its range along the southern shoreline of Lake Malawi (Alharbi et al., 2023). For National Control Programme activities, aggregated disease prevalence, arising from a handful of sampled primary schools, demonstrates that the locally dominant form, urogenital schistosomiasis, is sufficient to warrant annual mass drug administration of praziquantel across all primary schools in Chikwawa. With the recent revision in the World Health Organization 2022 guideline on control and elimination of schistosomiasis (WHO, 2022), current preventive chemotherapy strategies form the present minimum public health intervention. It will, however, need intensification in future as each form of schistosomiasis will likely increase across communities; as with expansion of irrigation areas, new freshwater habitats for intermediate host snails (i.e. *Bulinus* spp. and *Biomphalaria* spp.) will arise, alongside new water contact risk-points, making increasing transmission of human and animal schistosomiasis likely. For example, the capacity for significant epidemiological change has been recently evidenced upon detection of a fulminating outbreak of intestinal schistosomiasis in Mangochi District. This has arisen from an expanding local distribution of *B. pfeifferi* to newly colonise the Lake Malawi and Upper Shire River shorelines (Alharbi et al., 2023).

## 4 Integrated vector surveillance and control in emerging agricultural systems

Maintaining the socio-economic benefits of the SVTP without accentuating infectious disease will need ideas and perspectives from across ecological, veterinary, medical, and environmental science. This One Health approach (Cunningham et al., 2017) recognises that the health of small-holder farmers, their families, and the wider community in Chikwawa is inextricably linked to the wildlife, livestock and environment. Learning how people, animals and wildlife interact within the context of the SVTP can lead to locally appropriate control solutions that not only prevent vector-borne disease but also benefit animal and environmental health. The primary goal of the Shire Valley Vector Control Project (Shire-Vec) (https://www.lstmed.ac.uk/shire-vec) is to understand the impact of newly irrigated land on vector-borne diseases in the lower Shire Valley and provide locally appropriate solutions that mitigate any adverse effects. Box 1 presents several research questions across multiple disciplines pertinent to achieving this goal. Alongside the research programme, Shire-Vec will engage in discussions among various stakeholders from both the agricultural and public health sector in Malawi to promote the benefits of taking a multi-disciplinary approach to vector control as well as conduct a policy analysis to understand how vector control can be integrated within national agricultural and irrigation policy guidelines. Sustained top-down vector control interventions have contributed to substantial reductions in malaria and schistosomiasis case burden across Malawi over the past 15 years. There was a 47% reduction in malaria parasite prevalence in children between 2 and 10 years of age between 2010 and 2017 (Chipeta et al., 2019; Chirombo et al., 2020) and although the country did not reach 2021 global strategic targets, malaria incidence has continued to fall (WHO, 2022). There is a growing recognition, however, that locally tailored and more sustainable solutions are needed to break the transmission cycle of vector-borne diseases. This is particularly the case in irrigated farmland where disease risk may be higher. One promising solution to prevent vector populations from establishing in irrigated land is water and environmental management. When designed with public health concerns in mind, water management offers the potential to address several health issues at the same time (Boelee et al., 2019). The spillover of stagnant water from the main and supplementary canals, overflow from irrigation systems such as sprinkler, furrow, or pivot irrigation and improper drainage can all lead to favourable ecological habitats for vector populations. Environmental management interventions include the installation, cleaning, and maintenance of drains, vegetation management, promotion of water flow, intermittent irrigation, and systematic elimination of standing pools of water (Keiser et al., 2005). For example, the manipulation of water levels can modulate the flow and depth of the aquatic vector habitat, preventing mosquito oviposition. Historically, environmental management has reduced the burden of malaria and schistosomiasis in Africa and across the world (Keiser et al., 2005; Wilson et al., 2020), but the adoption of insecticide-based vector control and clinical interventions has less to a reduction in its use. The adoption of such an approach in the catchment of the STVP would not necessarily replace existing frontline interventions but add to the resilience of the ecosystem against vector populations.

Box 1 **A list of primary research questions within the Shire-Vec project to address the impact of agricultural transformation on vector-borne disease.**

***Entomology and malacology***

• What impact does the creation of newly irrigated land have on seasonal and long-term mosquito and snail biology and population dynamics?
• Are different irrigation types or cropping systems more favourable for invasive mosquito and snail populations?
• What is the evidence for arboviral transmission in the Shire Valley?

***Epidemiology***

• How much variability and temporal dynamics are there in prevalence of malaria and schistosomiasis within and between schools, and what are the key environmental drivers, taking into account existing control interventions?
• What are the knowledge gaps in the basic epidemiology of co-infection of urogenital and/or intestinal schistosomiasis with malaria, and once identified can these be exploited for better integrated control?
• What is the extent of zoonotic transmission of schistosomiasis?
• Over small spatial and temporal scales, how well do observed patterns in school-level prevalence reflect ongoing general patterns within associated communities?
• How will human water contacts and water, sanitation and hygiene (WASH) change during expanded irrigation initiatives, and will this (de)focalise the environmental transmission of schistosomiasis more generally?

***S*** ***ocial anthropology***

• How do communities frame permanent resettlement and what priorities do they have?
• How are daily and seasonal practices affected by irrigation as a structural intervention?
• What are the perceived effects of the SVTP on the local ecology?
• What effects will the scheme have on access to labour?
• What are the consequences of irrigation as a symbolic intervention on perceptions of family, social and moral duties, intrahousehold power relationships, daily and seasonal activities, and wellbeing?
• How is the land going to be organised and what are the cultural and practical aspects of land (re)distribution?
• What are the community perceptions of the environmental changes and new relationships with the land?

***Vector control***

• What is the impact of water and environmental management on entomological parameters and crop yield?
• Do house modifications lead to reductions in house entry of mosquitoes and are these acceptable to communities?
• Do spatial repellents prevent outdoor biting in farming communities?

***Modelling vector-borne disease transmission***

• What is the variation in malaria burden associated with environmental and land use changes in the lower Shire Valley?
• Can we use mathematical modelling approaches to infer vector abundance and transmission dynamics using available data?

Alt-text: Box 1

## 5 Conclusions

The primary purpose of irrigation is to alleviate uncertainty for the grower. The benefits of exploiting natural water sources for staple and cash crops are unequivocal, yet like so many anthropogenic endeavours, there are potential environmental and livelihood trade-offs. By harvesting existing freshwater sources, irrigation redistributes and shifts the nature of aquatic habitat. Lessons from primary research and systematic reviews conducted on vector-borne disease systems elsewhere in sub-Saharan Africa demonstrate the direct biological impact of irrigation on insect vectors and disease transmission (Steinmann et al., 2006; Kibret et al., 2021). To get closer, however, to a truer and more intuitive understanding of the impact of irrigation on vector-borne diseases, we must consider the local environmental, economic, and societal context. This requires - to name just a few factors - data on local disease transmission dynamics, community structures, the predominant vector populations and their behaviour, the degree of contact between farmers and families with both potential livestock and wildlife hosts, housing structures, and the existence and uptake of vector control. We propose that the phased development of the SVTP provides the opportunity to take such a multi-disciplinary approach and prevent any exacerbation of endemic or the emergence of epidemic vector-borne disease in the lower Shire Valley associated with such land-use change.

## Funding

This article is funded by the NIHR [NIHR Global Health Research Group on Controlling Vector Borne Diseases in Emerging Agricultural Systems in Malawi (NIHR133144)/NIHR Evaluation, Trials and Studies Coordinating Centre (NETSCC)]. The views expressed are those of the author(s) and not necessarily those of the NIHR or the Department of Health and Social Care.

## Ethical approval and consent to participate

Not applicable.

## CRediT authorship contribution statement

**Christopher M. Jones:** EiC: Revert to EDIT 2: Delete space before colon and add at the end "All authors read and approved the final manuscript."Conceptualization, Writing – original draft, Writing – review & editing, Funding acquisition. **Anne L. Wilson:** Conceptualization, Writing – review & editing, Funding acquisition. **Michelle C. Stanton:** Conceptualization, Writing – review & editing, Funding acquisition. **J. Russell Stothard:** Conceptualization, Writing – review & editing, Funding acquisition. **Federica Guglielmo:** Conceptualization, Writing – review & editing, Funding acquisition. **James Chirombo:** Conceptualization, Writing – review & editing, Funding acquisition. **Lindiwe Mafuleka:** Conceptualization, Writing – review & editing, Funding acquisition. **Rose Oronje:** Conceptualization, Writing – review & editing, Funding acquisition. **Themba Mzilahowa:** Conceptualization, Writing – original draft, Writing – review & editing, Funding acquisition. All authors read and approved the final manuscript.

## Declaration of competing interests

The authors declare that they have no known competing financial interests or personal relationships that could have appeared to influence the work reported in this paper.

## Data Availability

Not applicable.
